# A Digitization Framework for Belt Rotation Monitoring in Pipe Conveyor Applications

**DOI:** 10.3390/s25216792

**Published:** 2025-11-06

**Authors:** Leonardo dos Santos e Santos, Paulo Roberto Campos Flexa Ribeiro Filho, Emanuel Negrão Macêdo

**Affiliations:** 1 Graduate Program in Natural Resources Engineering of the Amazon (PRODERNA), Institute of Technology (ITEC), Federal University of Pará (UFPA), Belém 66075-110, Brazil; enegrao@ufpa.br; 2 Research Asset Management, Lab Equipment Management (LEM), King Abdullah University of Science and Technology (KAUST), Thuwal 23955-6900, Saudi Arabia; 3 Mechanical Engineering Department, State University of Maranhão (UEMA), São Luís 65055-310, Brazil; paulofilho@professor.uema.br

**Keywords:** pipe conveyors, belt rotation, digitization, physical prototype, Industrial Internet of Things

## Abstract

Pipe conveyors provide an environmentally friendly alternative to open-troughed bulk solids conveyance, particularly for long or complex routing applications. However, the sustainability of this technology is compromised by unstable operations. Complex routing, operational variations, and environmental factors create uneven contact forces, triggering belt rotation. This is a critical failure mode that requires continuous monitoring throughout the conveyor’s lifecycle. Insufficient failure data represents a typical challenge for this application. This study hypothesized technological principles that constitute the minimum requirements for enabling the scaling of industrial applications of belt rotation monitoring. Enabling technologies were adopted to foster innovation, and a physical prototype was implemented to address data scarcity for this failure mode. Using a controller-responder wireless network of ESP32 Industrial Internet of Things devices, we developed a belt-independent measurement system with multiparameter capability. Key criteria for detecting unsafe operational states and a criticality-based approach for determining optimal measuring unit quantities were established. The measurement results demonstrated suitable precision for digitization objectives: overlap angle (3.3107° ± 16.7562°), pipe diameter (+13.3850 ± 7.2114 mm), and overlap length (−26.2750 ± 25.1536 mm), based on 307 samples with a latency of 350.1303 ms. The framework demonstrates potential for industrial deployment with acceptable performance for real-time monitoring.

## 1. Introduction

Under ideal conditions, a pipe conveyor belt should run with overlap positions at the top and bottom on the carry and return sides, respectively, for all loading conditions. In addition, no belt rotation should occur during the conveyor’s startup or inertial stopping. In real-world applications, however, complex routing, operational variations, and environmental externalities lead to uneven forces of contact, triggering position changes that may exceed the tolerable operational limits. The consequences include mistracking the belt on conveyor pulleys, collapse of the tubular shape, or permanent operation under suboptimal circumstances. Impacts range from incident-based events, such as those related to belt rip, ceased production, or loss of profit, but also encompass recurring costs, such as belt replacement due to accelerated aging; lifecycle costs, such as failure-finding tasks, overlap monitoring, idler replacement, and energy loss; risk-related costs, such as insurance coverage and the organization’s image [1].

The belt rotation in a pipe conveyor is a critical failure mode, caused by a complex nonlinear interaction between various failure mechanisms, for which overlap position monitoring is required throughout the conveyor’s lifecycle. Typically, adopting this technology to the detriment of traditional open-troughed belt conveyors involves long or complex routing, demanding regular inspections along the conveyor’s flight and tracking the overlap position over time to map belt rotation or twisting phenomena [1]. Here, we adopt the term “twist” to refer to belt rotations that accumulate one or more absolute turns. Belt rotation affects the performance and reliability of pipe conveyors throughout their entire lifespan [2,3].

Pipe conveyor operation is a mission-critical application, and next-generation digital technologies have the potential to enhance digitization through the integration of cyber–physical twins, the Industrial Internet of Things (IIoT), and assisted computing using generative artificial intelligence (GenAI). Monitoring belt stability requires recurring human intervention, and insufficient data is a typical scenario given the number of dependent variables with highly nonlinear characteristics. According to Richey Jr et al. [4], such scenarios require high GenAI capabilities, with supervised machine learning proposed as an approach for context-unknown logistics and supply chain management applications. Fedorko et al. [5] highlighted the role of digitization in supporting the monitoring of dynamic, static, and combined undesirable operational conditions. Their experimental study focused on the asymmetry of contact force in the transition zone of pipe conveyors.

Given the operational characteristics of pipe conveyors, learning temporal and spatial patterns predominantly from belt rotation incidents is impractical or intolerable from engineering and risk management perspectives. Although this technology, originally designed and patented in 1979 by Kunio Hashimoto [6], has successfully provided sustainable solutions worldwide, the digital monitoring of overlap positions remains unexplored. Equipment custodians frequently encounter challenges in maintaining a stable overlap position throughout the conveyor’s lifecycle, particularly during belt recommissioning following a repair or replacement.

### Operational Challenges in Pipe Conveyors

Off-the-shelf [7,8,9] and publicly available patented solutions [10,11,12,13,14,15,16,17,18,19,20,21,22,23,24,25,26,27,28,29,30,31] mainly propose a failure-finding approach based on belt rotations beyond predefined thresholds. Zhao et al. [32] developed a deviation detection system for pipe conveyors based on image processing. They proposed an innovative shape-prior learning architecture for detecting the overlap beltline and assessing pre-defined thresholds in half the circumference of the pipe shape. For twisted belts, this condition can lead to misinterpretations in monitoring systems that rely solely on angle detection, posing a challenge for both human-based and digitized inspections. In addition, monitoring solutions that rely on physical components manufactured with or inserted along the belt cover, such as magnetic markers or inductive sensor loops, may be damaged by seized rolls or contact with structures or deactivated due to the replacement of minor belt sections, resulting in limited maintainability.

Therefore, developing a digitization framework is of research interest, enabling further adoption of synthetic or augmented data approaches for assisted computing using cyber–physical twins. To this end, we hypothesized six technological principles as a minimum to enable large-scale industrial application of belt rotation monitoring in pipe conveyors. In our proposed framework, the prototype aims to create an intermediate physical twin to address the data and labeling scarcity of the belt rotation failure mode, thereby mitigating its effects in real-world applications. By incorporating field variables, we aim to bridge the gap in training data for assisted computing and enhance the technology readiness level (TRL) of existing monitoring methods. In this study, we designed and implemented a multiparameter prototype of a pipe conveyor belt section by using a dedicated test apparatus. This was achieved by reproducing and expanding the method proposed by dos Santos e Santos et al. [33], given its first-of-its-kind multiparameter implementation of a cyber–physical twin for monitoring belt rotation in pipe conveyors. To this end, we adopted three enabling technologies to support this digitization approach.

In the physical layer, we adopted analog IR distance sensors connected to ESP32 IIoT devices and employed a controller-responder wireless network. Our expanded approach comprised an adjustable positioning mechanism for the measuring device and a variable-speed vibration motor. We also implemented mechanisms to regulate the belt-to-diameter ratio (B/D ratio) and switch the belt’s outer edge position. A movable stand equipped with a handle was used to rotate the belt sample. Bluetooth sensor modules were implemented to collect 3-axis motion, vibration, and room temperature measurements. We implemented an edge layer using Python, then fine-tuned an LLM, prompting structured keywords to its attention mechanism, along with distance measures, to predict the angle of the belt overlap and the belt’s outer edge position. Assisted computing was performed on OpenAI servers via an application programming interface (API).

We then developed a human–machine interface (HMI) embedded with data labeling and logging features for training and data historicizing purposes. We successfully reproduced the methods to measure the overlap angle, angular speed, number of absolute turns, pipe diameter, and the belt’s outer edge position. Additionally, our expanded method incorporated the measurements of vibration in velocity (in mm/s RMS), room temperature (°C), illuminance (lx), overlap length (in mm), B/D ratio, and latency (ms).

## 2. Materials and Methods

This section presents the systematic approach adopted to implement the proposed framework. We designed and developed a test rig equipped with a belt sample, utilizing a sampling unit to validate the first five hypothesized technology principles. Then, we proposed a criticality-based mathematical model to implement multiple sampling along a pipe conveyor route and demonstrated the calculations in a real-case application.

### 2.1. Digital Transformation Context

The digitization of belt rotation monitoring in pipe conveyor applications poses significant technological challenges for integrating cyber–physical systems (CPS), IIoT, and GenAI. For instance, the time discrepancy between digital and physical twins could render the framework unfeasible. Frasheri et al. [34] proposed a co-simulation approach using a digital shadow as a mechanism to detect networking degradation through a case study involving a miniature robot equipped with an indoor positioning system.

In the context of IIoT, latency in data processing is a variable of concern. Liu et al. [35] highlighted latency issues in traditional architectures dealing with industrial data for cloud storage and processing. The authors proposed the adoption of an IIoT cloud–fog hybrid network (ITCFN) framework and validated their algorithm in a case study of a coke-pushing machine by collecting start–stop signals, positions, and images of push rods. Singh et al. [36] designed and implemented a system for machine monitoring, addressing various obstacles in integrating IIoT and artificial intelligence (AI) solutions to operational machines, with a higher impact on Small and Midsize Enterprises (SMEs). The custom-prototyped electronic board featured an ESP32 system-on-chip (SoC) module, employed the Message Queuing Telemetry Transport (MQTT) data exchange protocol, and an AI application rule engine for optimizing production planning in real-time.

Moreover, data or labeling scarcity may limit the use of assisted computing with GenAI for mission-critical industrial applications. In a case study utilizing a run-to-failure data repository, Hakami [37] investigated the impact of scarce failure data, imbalance in run-to-failure datasets, and dependency on mapping temporal patterns when applying machine learning techniques for predictive maintenance. The author generated synthetic data using a Generative Adversarial Network (GAN) [38] and deployed a Long Short-Term Memory neural network [39] to learn temporal patterns from the same components over time. Sobreira et al. [40] implemented a case study to address the scarcity of data in measuring mass flow in conveyor belts. The authors combined motor data of a target feed hopper with real data from a belt scale installed in a downstream conveyor, then successfully applied the machine learning method of Reduced Error Pruning Tree. In turn, Matos et al. [41] utilized a 2D Lidar for online recognition and classification of bulk solids properties transported via conveyor belts, including rock fragmentation and type. Li et al. [42] employed a data augmentation approach to overcome data scarcity in detecting failed wires in steel-cord-reinforced belts. Salim et al. [43] implemented an embedded UHF-RFID sensor in a fabric-reinforced belt to measure the received signal strength indicator in the event of belt cracks. Luo et al. [44] used the YOLOv5 computer vision model to analyze belt operation videos captured by an inspection robot, enabling the detection of contaminants in troughed belt conveyors. In another case study, Xu et al. [45] proposed a 3D point cloud-based method using a binocular laser camera to detect and characterize longitudinal rips in troughed belt conveyors.

In GenAI applications, the widespread adoption of autoregressive large-language models (LLMs) has led to multiple solutions for integrating industrial information. Tao et al. [46] conducted a case study using an LLM-based framework with public datasets for bearing fault diagnosis. The authors created a custom prompting approach based on time and frequency domain features for fine-tuning and output a standardized fault diagnosis classification. In addition, they adopted a reduced temperature to achieve higher stability, as well as an equal number of grouped samples for each failure mode to avoid class imbalance. They froze the attention layers to maintain the pre-trained knowledge of the selected model. Bahr et al. [47] proposed a knowledge graph-enhanced retrieval-augmented generation (KG-RAG) framework to enhance the capabilities in Failure Mode and Effects Analysis (FMEA). Their case study involved using a custom chatbot to query a comprehensive FMEA knowledge graph database. An LLM was used to preprocess user questions and post-process structured retrieval before providing answers. Specialist participants assessed the quality of the responses generated in terms of correctness, usability, relevance, completeness, and retrieval time. In a comprehensive study of AI’s impact on innovation management, Zhang and Zhang [48] highlighted that GenAI can improve the efficiency and quality of information integration in anomaly detection and risk mitigation.

Researchers and developers have employed various modeling and prompting techniques to mitigate the effects of class imbalance, model hallucinations, and uncertainty. Even for human-in-the-loop strategies, data annotation for pretraining can lead to a disagreement problem, where differences in opinion or belief between individuals or groups affect qualitative assessments [49].

Although autoregressive LLMs are increasingly accessible and integrated in industrial processes, the next-token approach poses a challenge in measurement applications. In general, directly outputting a float number representing a dependent variable in LLMs poses a complex challenge in addressing model generalization and loss function sensitivity during fine-tuning, which are not well-suited for continuous values. Yang et al. [50] introduced the Numerical Understanding and Processing Abilities (NUPA) benchmark to evaluate the numerical reasoning skills of LLMs. The benchmark organizes numeric concepts into different representations, including floating-point numbers, and covers tasks such as elementary arithmetic, comparison, digit recognition, and representation conversion. To provide a more detailed evaluation beyond exact match accuracy, the authors also measured digit match and length. Their results showed that models such as GPT-4o, GPT-4o-mini, and Qwen2 performed reasonably well on elementary tasks involving numbers of up to eight digits; however, their accuracy dropped significantly for larger digit lengths and more complex tasks.

### 2.2. Research Gap and Hypothesis

To address the challenges of digitizing belt rotation monitoring in pipe conveyor applications, we adopted a rationale for assessing the belt rotation failure mode and its effects to propose technological principles for governing large-scale industrial digitization efforts. The predominant operational scenario for pipe conveyors involves curved routing, which often technically justifies adopting this technology over traditional open-troughed belt conveyors. In pipe conveyors, the belt tends to rotate along curved flights [51], and belt tracking is not always effective, requiring extensive monitoring to support adjustments to tolerable limits [52]. This failure mode can lead to persistent downstream belt mistracking in the conveyor’s transition zones, with catastrophic consequences due to belt folding in the conveyor pulleys [1]. Therefore, every rotation beyond the tolerable limits has the potential to cause relevant operational and financial losses, posing a challenge to collecting labeled data from abnormal belt positioning and ultimately restricting the learning of temporal and spatial information from belt rotation events. Given these characteristics, we proposed adopting an intermediate physical twin to enable unrestricted simulation of rotation scenarios and address the data and labeling scarcity associated with the belt rotation failure mode. This resulted in our first technological principle: physical prototyping for training.

Next, we assessed off-the-shelf and publicly available patented solutions to identify favorable measurement techniques to support this digitization effort and enable large-scale industrial applications. Measurement approaches can be grouped into belt-independent or belt-dependent techniques. The former consists of non-contact measurements using distance sensors or image processing. Conversely, the latter involves embedding magnetic inserts, RFID tags at equidistant intervals along the belt during manufacturing or employing torque sensors in contact with the belt surface [1]. Here, maintainability is a key element when scaling monitoring applications. Pipe belts are prone to wear and tear, which can occur in various forms, including superficial, transverse, longitudinal, pierced, or at the belt edges. As a collateral effect, the loops embedded in the belt can be damaged or removed during operation or belt section replacements, requiring bypassing the monitoring systems. A similar scenario is recurring in the rip detectors employed in open-troughed belt conveyors. Additionally, the belt is subject to cyclical fatigue, contamination, aging, and weathering. For this reason, we proposed adopting belt-independent measurement techniques as the second technological principle in our digitization framework.

In pipe conveyors, belt rotation may be triggered by numerous factors, including environmental conditions such as strong winds, heavy rain, subzero temperatures, and cumulative exposure to sunlight, ozone, oxygen, and heat. Additional factors, such as prolonged shutdown, hard flowing, dewatered, or deteriorating materials, and unstable filling degrees, also contribute to this failure mode. Other aspects include improper routing, high belt speeds, belt sag, excessive belt cycling, improper belt reinforcement, low B/D ratio, improper transverse bending stiffness, poor structural alignment, heterogeneous hardness along the belt length, vulcanizing and splicing issues, excessive tension during installation, improper tension force, improper belt training, low tension in molding rolls, and missing or seized idler rolls [1]. As outlined by Zamiralova [53], this failure mode has yet to be explored, and practical belt-tracking approaches can be ineffective. Therefore, scaling solutions for rotation monitoring demands not only measuring the overlap position. Comprehensive data collection on auxiliary measures, including ambient temperature, light, vibration, and belt-to-diameter ratio, can support a predictive approach. We then proposed adopting multiparameter processing as the third technological principle in our digitization efforts.

**Figure 1:**
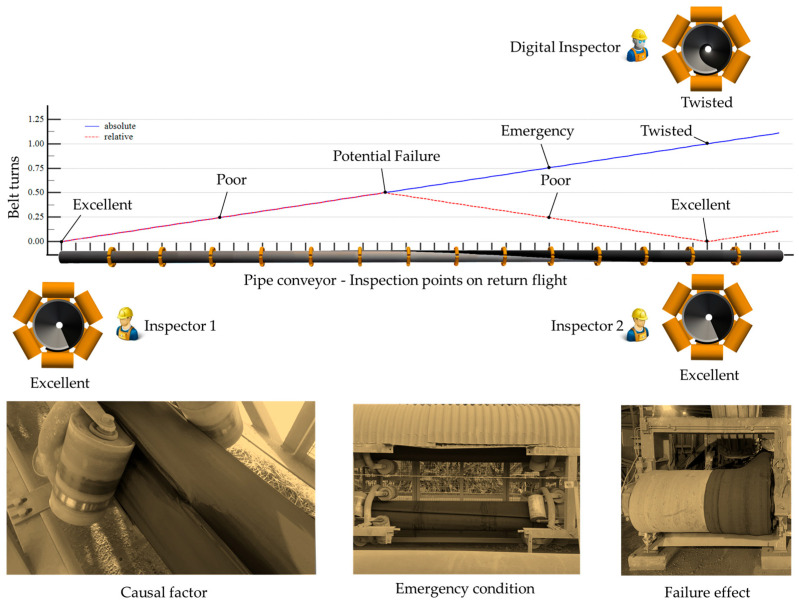
Misinterpretation problem in belt rotation monitoring. Outer edge alternance caused belt twisting and resulted in a functional failure due to belt folding in the pipe conveyor tail-end pulley. Images were recolored for academic purposes.

Furthermore, we evaluated the scenario of operational instability, which frequently affects pipe conveyor operations and can lead to twisted belts. Such scenarios can lead to misinterpretations in monitoring systems that rely solely on angle detection, posing a challenge for both human-based and digitized inspections. To exemplify the misinterpretation challenges caused by unstable or twisted belts, which frequently affect pipe conveyor applications, consider a hypothetical scenario involving two maintenance technicians inspecting a twisted belt on the conveyor’s return flight at two distinct points while communicating via radio, as illustrated in Figure 1. Given that the twisted belt appears at the bottom position at both inspection points, the technicians may erroneously conclude that the belt is in stable condition. Additional factors, such as access constraints, ambient lighting conditions, and human factors, can further contribute to this misinterpretation, potentially leading to delayed intervention decisions or operational incidents related to belt rotation. For this reason, we proposed the monitoring of absolute belt turns as the fourth technological principle in our digitization framework.

The first four technological principles unlock key operational variables for monitoring belt rotation in pipe conveyors. However, two critical challenges need to be addressed: identifying unsafe states and determining the time required to prevent the failure effect. The former demands a comprehensive understanding of predictive measures in pipe conveyor applications, including the target belt overlap position, the admissible number of turns, the critical belt rotation speed, or the optimal B/D ratio. The latter involves dealing with the system detection-response latency, the safe-stop time of the belt with controlled or inertial deceleration, and the length of the conveyor’s transition zone. In this sense, any scalable digitization effort for monitoring belt rotation must ensure the timely detection of unsafe states and prevent belt folding in the conveyor’s pulleys, which constitutes our fifth technological principle.

Finally, we considered the typical operational characteristics of pipe conveyors’ routing, which are often long or topographically complex. This demands frequent inspections along the route, with belt positioning recorded at critical points, mainly in curved flights. This knowledge management approach enables operational and maintenance teams to identify trends and anticipate undesirable scenarios. However, this is often based on unstructured decision-making due to a lack of labeled failure data. In this sense, we advocated adopting a unitary protection distance to estimate the number of devices required to mitigate the need for frequent human inspection satisfactorily. For this reason, we proposed multiple sampling along the route as the sixth technological principle for belt rotation monitoring in pipe conveyor applications, outlined in the following digitization framework:

- Principle 1: Physical prototyping for training.
- Principle 2: Belt-independent measuring techniques.
- Principle 3: Multiparameter processing.
- Principle 4: Monitoring absolute belt turns.
- Principle 5: Timely detection of unsafe states.
- Principle 6: Multiple sampling along the route.

In implementing our digitization framework, enabling technologies play a relevant role. In pipe conveyor applications, belt rotation monitoring can be significantly affected by conveyor routing, structural access, and manpower constraints [1]. Therefore, scalable solutions need to consider data integration in harsh, remote environments, while also prioritizing maintainability, which favors the adoption of IIoT networking. Additionally, implementation costs and time are sensitive variables, particularly for SMEs, and adopting LLM-assisted computing can support a fast-paced, scalable deployment. Further, the availability of real-time unsafe conditions or predictive metrics, along with data annotation capabilities, incentivizes the adoption of human–machine interfacing for belt rotation monitoring. Therefore, we adopted the following three enabling technologies in our digitization efforts:

- Enabling Technology 1: IIoT networking.
- Enabling Technology 2: LLM-assisted computing.
- Enabling Technology 3: Human–Machine Interfacing.

**Figure 2:**
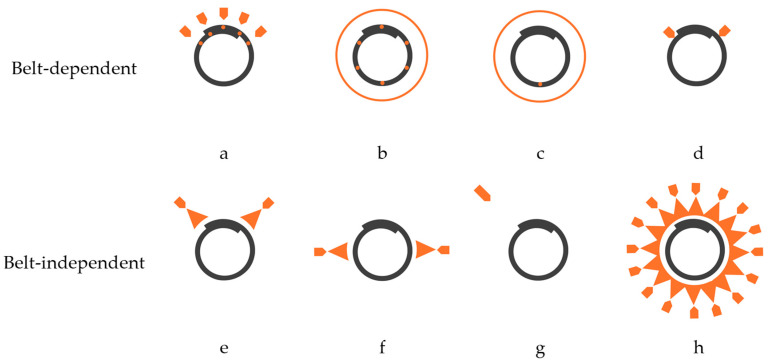
Typical measurement techniques for monitoring belt rotation in pipe conveyors: magnetic inserts and RFID tags at top [8] (**a**), magnetic inserts along the belt width [23] (**b**), magnetic inserts at the bottom [9] (**c**), torque sensors [54,55] (**d**), laser sensors [21] (**e**), lidar sensors [7] (**f**), image processing [56] (**g**), and the propose adoption of IR distance sensors (**h**).

To establish and assess the performance of our proposed digitization framework relative to existing methods, we defined target features. For publicly available patented solutions, the theoretical basis limits the assessment of measurement accuracy. In turn, off-the-shelf manufacturers do not disclose their system metrics, and the industrial adoption of rotation monitoring solutions in pipe conveyors has yet to be explored [33]. At a conceptual level, our target features include measures for: the relative and absolute positions of the belt overlap, the belt rotation speed, and the B/D ratio. We then assessed seven typical measurement techniques outlined in the existing methods. We concluded that only the absolute position of the belt overlap is a standard measurement feature, which favors our proposed framework as a promising approach for large-scale application of belt rotation monitoring in pipe conveyors. Figure 2 illustrates the typical measurement techniques adopted for belt rotation monitoring.

### 2.3. Implementation of Technological Principles

In this study, we designed a vertical network architecture comprising physical, edge, and cloud layers. The physical layer consisted of a theoretical pipe conveyor belt and a real physical prototype, which constituted the observable physical elements. The edge layer comprised an IIoT controller and an HMI, while the cloud layer provided computational resources for assisted computing. Additionally, we established a horizontal architecture that divided the physical and cloud layers between training and inference functions, with the cloud layer operating as a switching mechanism through the implementation of a digital twin framework.

For digitization, the method involved switching to a digital twin and using its HMI to collect labeled data from the physical prototype in the physical layer. The cloud layer was then configured, in the vertical architecture, to fine-tune an LLM using these collected samples. This approach composed the training function in horizontal architecture.

**Figure 3:**
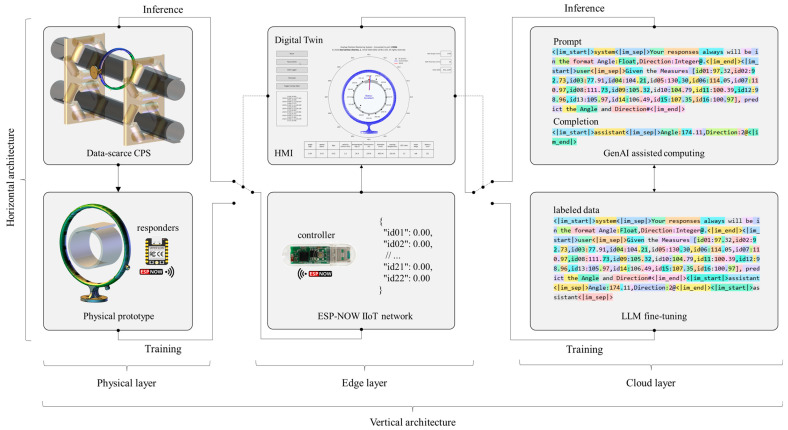
Network architecture for digitization of belt rotation monitoring.

Next, the HMI switched the digital twin to collect data from the operational application in the physical layer while configuring the cloud layer to perform inference using the fine-tuned LLM, thereby enabling monitoring and analysis of belt rotation data. This approach composed the inference function in a horizontal architecture. Figure 3 illustrates the network architecture for digitizing belt rotation monitoring.

#### 2.3.1. Principle 1: Physical Prototyping for Training

In our implementation, we created an intermediate physical twin to address the data and labeling scarcity associated with the belt rotation failure mode. We designed a hexagonal wooden frame with an apothem of 170 mm, a thickness of 7 mm, and a height of 100 mm. Two wooden disks, each measuring 152 mm in diameter and 12.7 mm thick, were attached to the hexagonal frame and hollowed to accommodate a 300 mm × 16 mm shaft, which was secured using a rigid flange coupling. We then attached the shaft to a movable on-the-shelf stand using a UCF202-16 bearing (FYH Bearing Units, Osaka, Japan), which is a 16 mm bore, 4-bolt square-flanged, cast iron self-lubricating housed bearing. A manual lever was attached to the end of the opposed shaft. The stand was a floor-mounted swivel type with the shaft centerline positioned 970 mm above the floor surface.

To regulate the B/D ratio belt, we attached lifting platforms to five of the six outer faces of the hexagonal frame, each with a 90 mm × 90 mm surface area and height adjustable from 30 to 90 mm. We printed and attached curved shims in PLA with a radius of 180 mm and a maximum height of 10 mm to the lifters’ surfaces to smooth the belt conformation into a tubular shape. We also attached a 360° analog angle finder for visual reference at the 12 o’clock position. To collect 3-axis motion measurements, a Bluetooth sensor module, WT901BLECL (WitMotion, Shenzhen, China), was attached at the 6 o’clock position. This battery-powered module features a 250 mAh battery capacity with a Type-C charging connection, an inclinometer accuracy of 0.05° on both the x and y axes, and a 16-bit gyroscope resolution with a stability of 0.05°/s. A 5000 mAh power bank was attached to the wooden frame and connected to the module using a 300 mm on/off Type-C extension cable.

**Figure 4:**
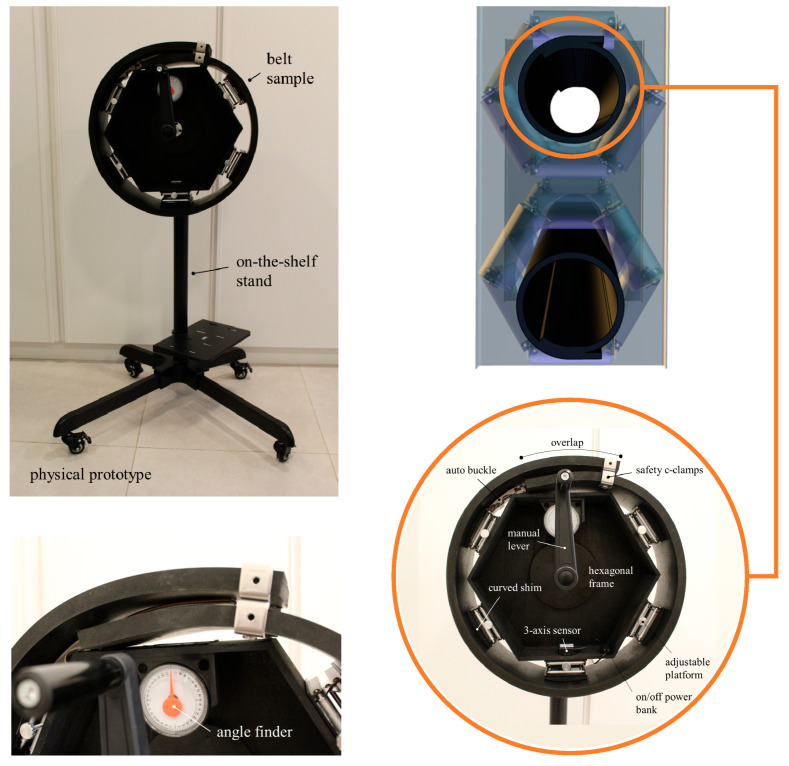
Physical prototype.

We used a black neoprene and styrene butadiene rubber (SBR)-blended sheet for the belt sample, which measured 1700 mm in length, 20 mm in thickness, and 150 mm in width. We applied an auto-lock buckle and safety stainless steel C-clamps to secure the B/D ratio and maintain its tubular shape. The sample had a specific gravity of 1.70 ± 0.05 and a hardness of 70 ± 5 Shore A. Its mechanical properties include a minimum tensile strength of 35 kg/cm^2^, a minimum elongation at break of 200%, a maximum compression set of 50%, and a minimum angular tear resistance of 15 kg/cm. The blended sheet contained 10% neoprene polymer and demonstrated good resistance to ozone, as well as diluted acids and bases. However, it is not recommended for use with concentrated acids, bases, oils, or solvents. Heat aging tests resulted in a maximum hardness change of 5 points, with changes in tensile strength and elongation at break ranging from +10% to −25%. The operational temperature range spans from −30 °C to +70 °C. Figure 4 shows the physical prototype of the proposed system. By releasing the auto-buckle and safety C-clamps and adjusting the five lifting platforms, different B/D ratios can be set. The manual lever and stand swivel enabled the reproduction of multiple belt rotation scenarios, providing training data that would be unachievable in a real-world application.

#### 2.3.2. Principle 2: Belt-Independent Measuring Techniques

To implement a non-contact measurement system, we designed and printed a 16-part circular frame in PLA with a diameter of 775 mm and an elliptical cross-section measuring 88 mm and 38 mm along the major and minor axes, respectively. The 16 parts were hollowed with a wall thickness of 4 mm. Each part was provided with an embedded analog output-type IR sensor for distance measuring, model Sharp GP2Y0A21YK0F (Sharp Corporation, Osaka, Japan), whose measuring range varied from 100 to 800 mm. This sensor operates within a voltage range of 4.5–5.5 V, uses a 3-pin JST PH connector, and features an update period of 38.3 ± 9.6 ms. We connected the sensors to Seeed Studio XIAO ESP32C3 IIoT (Seeed Studio, Shenzhen, China) development boards and powered the modules using a 20-port USB hub, which was wired via USB-A to USB-C cables.

**Figure 5:**
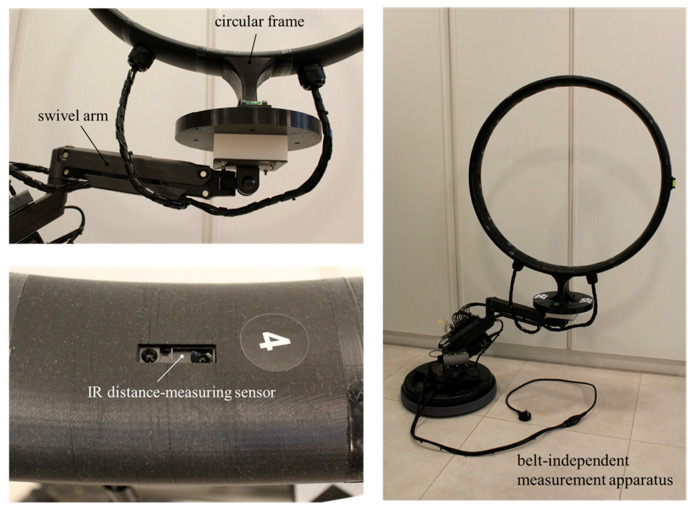
Belt-independent measurement apparatus.

Next, the measuring frame was attached to an off-the-shelf full-motion gas-spring swivel arm. A 20 kg rubber-coated weight plate was used to stabilize the assembly. The assembly was placed on a 40.3 cm movable plastic round tray. A 90 W, 12 V, 7.5 A power supply was attached to the arm frame, along with additional components used for multiparameter processing. Figure 5 shows the belt-independent measurement module. The full motion of the apparatus allowed for adjustable positioning relative to the belt sample, thereby expanding the capabilities necessary for further industrial implementation.

#### 2.3.3. Principle 3: Multiparameter Processing

In our implementation, we added a variable-speed SL-3650C Series vibration motor (Shanglinmotor Automations, Dongguan City, China) to simulate the excitation frequencies from the belt and idler rolls, as expected in real-world applications. The device was a double-headed eccentric wheel type, powered by 12 V, with a maximum efficiency of 168.08 g.cm at 3148 RPM. It was attached to the arm frame, along with a 24 W, 3–12 V, 2 A adjustable power supply.

Next, we attached a Bluetooth sensor module, WTVB01-BT50 (WitMotion, Shenzhen, China), to the arm frame. This battery-powered module was used to measure the vibration velocity and room temperature. It features a 260 mAh battery capacity and a Type-C charging connection. The detection cycle and cutoff frequency were 1–100 Hz, with an operating temperature range of −20 to 60 °C. It was connected to a 20-port USB hub via a USB-A to USB-C cable.

Moreover, we customized a Bluetooth-to-ESP-NOW interface to integrate Bluetooth devices into the IIoT network. The employed ESP32 devices (Seeed Studio, Shenzhen, China) shared Bluetooth and Wi-Fi protocols using a single antenna in coexistence mode via coordinated access. However, to achieve low latency in our application, we opted to use a pair of Seeed Studio XIAO ESP32C3 IIoT development boards to process the Bluetooth and Wi-Fi connections separately. This was achieved by interconnecting the universal asynchronous receiver–transmitter (UART) and power pins on paired ESP32 boards without the need for additional wiring. Each pair was directly connected to a 20-port USB hub using USB-A to USB-C adapters.

**Figure 6:**
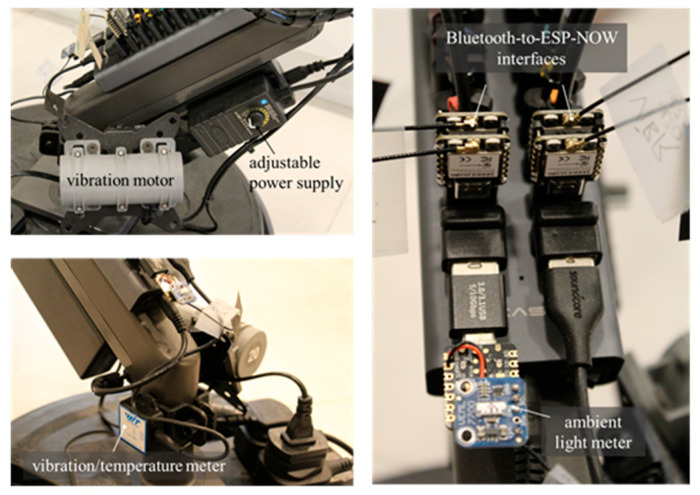
Multiparameter measuring devices.

We then integrated a 16-bit ambient light sensor VEML 7700 (Vishay Intertechnology, Malvern, PA, USA) into a Seeed Studio XIAO ESP32C3 IIoT development board, utilizing the I^2^C communication protocol to measure illuminance. A USB adapter (A to C) was employed to connect the assembly directly to a USB 20-port hub while keeping the light transducer facing up at 54°. Figure 6 shows the variable-speed vibration motor, customized Bluetooth-to-ESP-NOW interfaces, and the ambient light measuring board.

#### 2.3.4. Principle 4: Monitoring Absolute Belt Turns

In our study, the number of belt turns was calculated by measuring angle variations over two consecutive measurement cycles. We assumed that the measurement latency was much smaller than the time required for a significant belt rotation in real-world applications. Here, we adopted the prior assumptions employed by dos Santos e Santos et al. [33], who considered a 180° belt rotation at 10 RPM as the potential failure threshold owing to belt rotation. Therefore, we calculated the relative number of turns as a 2-digit float resulting from the measured or predicted angle divided by 360°, assuming the target position as the top overlap position (0°). To compute the absolute number, we adopted incremental and decremental values for clockwise and anticlockwise rotations, respectively, in relation to the belt’s running direction. We implemented an algorithm to detect when the readings crossed the boundary between 0° and 359.99°. When the variation between consecutive cycles was less than −300° (e.g., from 357° to 2°, resulting in −355°), the rotation was deemed clockwise, and we incremented the turn counter by 1 unit; conversely, when the variation exceeded +300° (e.g., from 1° to 359°, resulting in +358°), the rotation was deemed anticlockwise, requiring decrementing the turn counter by 1 unit.

**Figure 7:**
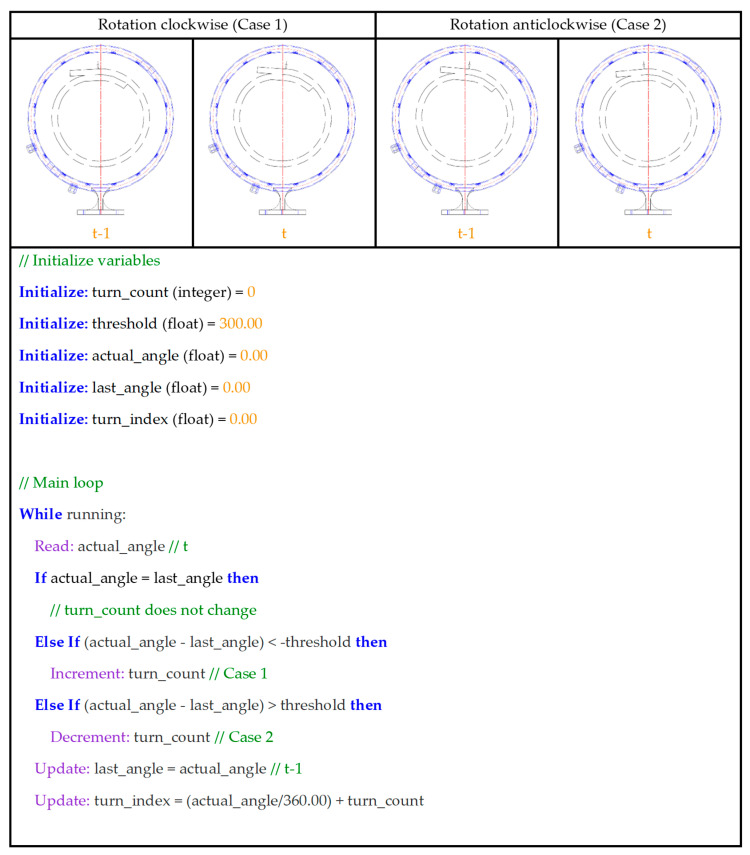
Pseudocode for monitoring the absolute number of belt turns.

We implemented these calculations in the network and application layers. The former was employed in the 3-axis motion Bluetooth-to-ESP-NOW interface, specifically the Bluetooth board, to provide training data. The latter was applied to post-process the predicted angle and compute the absolute number of turns required. During inference, we used a weighted moving average filter for the five most recent measurements, with weights ranging from 0.5 to 1.0, which favored newer data. Additionally, we implemented a multithreaded execution to maintain system responsiveness while predicting the overlap angle and the belt’s outer edge position. The adoption of a weighted moving average penalizes measurement synchronization for the benefit of enhanced LLM hallucination mitigation and inference stability. Figure 7 presents a pseudocode of the adopted approach for monitoring the absolute belt turns.

#### 2.3.5. Principle 5: Timely Detection of Unsafe States

In the application layer, we implemented four predictors of unsafe states: absolute number of turns, B/D ratio, overlap length, and the belt’s outer edge position. We added numeric fields to be populated by the system user with the sample length (belt width) and thickness (mm). We then set the default values to 1700 and 20 mm, which corresponded to the belt sample used in our experiment. Additionally, we utilized an HMI button to toggle the predefined position of the outer edge of the belt.

First, we applied the criterion originally proposed by dos Santos e Santos et al. [33], which uses the number of turns to indicate the operational stability of the pipe belt as follows: excellent (less than 1/24 turn in either direction), good (between 1/24 and 1/12 turns), fair (between 1/12 and 1/8 turns), poor (between 1/8 and 1/4 turns), critical (between 1/4 and 1/2 turns), and potential failure (more than 1/2 turns). This was applied to both clockwise and counterclockwise rotations. Using the sample length parameter (which represents the belt width, B) and the calculated pipe belt diameter (diameter, D), we computed the B/D ratio. Typically, this ratio ranges between 3.5 and 4 [51,57,58,59,60] and can be a good predictor of troughability when compared to the designed or usual operational values. This was achieved by processing 16 distance measures to calculate the pipe diameter. First, we converted the radial distances of the sensor into Cartesian coordinates, considering the device’s radius. Next, we applied least-squares optimization to estimate the center of the belt sample. We then determined the distances between pairs of opposite sensors (e.g., at 0° and 180° or 45° and 225°). Subsequently, we ranked the eight paired values and filtered potential outliers by dismissing the first and last three values. Finally, we applied a 20-sample moving average to ensure measurement stability.

Additionally, measuring the diameter of the belt throughout the lifecycle of a pipe conveyor is a recommended practice. It can also be a good predictor of low troughability during commissioning or excessive tension after the troughing phase. Here, we adopted the empirical assumptions proposed by dos Santos e Santos et al. [33], in which a 15% reduction in diameter of the belt represents a potential failure threshold owing to increased troughability. It is worth mentioning that the loss of contact forces between the belt and idler rolls, as well as operational stability, are key factors in the analysis and must be considered together by specialist engineers.

Next, we calculated the overlap length, which is a good predictor of suboptimal belt tension. Using the sample length and thickness parameters, along with the measured angle and calculated diameter, we implemented a 1000-step spiral approximation. We used the angle between the start and end of the spiral function to determine the arc length of the overlapping section. Furthermore, we employed Cartesian coordinate transformations and least-squares optimization for positioning the spiral at the Cartesian center. Then, we shifted its position using the estimated center of the belt sample to provide a dynamic visual reference in the HMI.

Finally, we monitored the position of the outer edge of the belt by encoding it as integer values: undefined (0), right-sided (1), or left-sided (2). This is a good detector of overlap alternation resulting from a low belt tension. We implemented this calculation during training in the application layer, using the preset belt outer-edge position as specified in the HMI. We applied a buffered filter to the five most prevalent measurements during the inference process. Table 1 summarizes the key criteria for detecting unsafe states related to belt rotation in pipe conveyors.

#### 2.3.6. Principle 6: Multiple Sampling Along the Route

In our proposed framework, we theorize the implementation of multiple sampling points along the route of a pipe conveyor to prevent functional failures due to belt rotation. We proposed at least two measuring devices positioned upstream of the drive and return pulleys, to protect the carry and return transition zones of the pipe conveyor as a minimum. These are critical regions where the belt transitions to or from its enclosed shape, guided by reinforced molding rolls of highly concentrated stress on the pipe belt carcass.

We then assign a factor k to differentiate between criticality levels: 2 for long or topographically complex routes and 1.5 for ordinary or highly stable routes. This approach supports the determination of an optimal and cost-effective number of measuring devices. It shall be assessed by designers and specialist engineers, considering project criteria, technical constraints, and operational expertise. This factor indicates how to split the devices between the carry and return flights at a 1:1 and 1:1/2 ratio, respectively.

The rationale for determining the unitary protection distance was to establish a target position for the measurement device, ensuring the belt can safely stop before the rotated section reaches the conveyor’s transition zone in the event of an abnormality. Therefore, four distances were considered. First, the beginning of the longest transition zone was adopted as a conservative target position for ending a safe belt stop. Next, three additional distances were defined based on the belt’s scalar speed and time components. The first component corresponds to the time required for the belt to rotate from the target to a non-tolerable position, expressed in terms of the admissible number of turns and the critical belt rotation speed. The second component represents the detection-response time, that is, the time from anomaly detection until the conveyor begins the safe-stop process. Finally, the third component corresponds to the belt’s safe-stop time under controlled or inertial deceleration.

To calculate the unitary protection distance, we considered the most extended transition zone on the conveyor, the nominal belt speed, the admissible number of turns, the safe-stop time with controlled or inertial deceleration, and the system’s detection-response latency. The smallest successive integer resulting from the division of the developed conveyor length and unitary protection distance indicates the number of devices required, as expressed in (1).

(1) $$n_{u}=\max\left\{2,\;\left\lceil k\left[\frac{L}{T_{z}+{\;V}_{b}\left(\frac{60\;n_{a}}{\omega_{r}}+\tau_{dr}+\tau_{s}\right)}-1\right]\right\rceil \right\}$$

where $n_{u}$ is the recommended number of measuring units; k is the criticality factor; L is the developed conveyor length, in m; $T_{z}$ is the length of the most extended transition zone, in m; $V_{b}$ is the nominal longitudinal speed of the belt, in m/s; $n_{a}$ is the admissible number of turns; $\omega_{r}$ is the critical belt rotation speed, in RPM; $\tau_{dr}$ is the detection-response latency, in s; and $\tau_{s}$ is the safe-stop time with controlled or inertial deceleration, in s.

### 2.4. Implementation of Enabling Technologies

To support the implementation of the proposed network architecture, we adopted three enabling technologies to interconnect the physical, edge, and cloud layers. We employed IIoT networking to enable the data collection from the observable physical elements to the digital twin. We then applied LLM-assisted computing to train on the physical prototype data and predict the overlap angle and position of the outer edge of the belt. In turn, we developed an HMI to create a digital twin of the physical entities and to switch the network horizontal layer between training and inference functions.

#### 2.4.1. Enabling Technology 1: IIoT Networking

For IIoT networking, we employed the ESP-NOW wireless communication protocol to achieve multiparameter processing and create a deployable sampling unit. We specified a USB IIoT dongle, model LILYGO T-Dongle S3 ESP32-S3 Development Board (LILYGO, Shenzhen, China), as the network controller for the system. The network architecture consisted of one controller and 19 physical nodes, configured as responders. A total of 22 SoCs were employed in the physical layer, owing to the paired ESP32 approach used to measure vibration and 3-axis motion using our custom Bluetooth-to-ESP-NOW interfaces. In the network layer, 22 variables were created.

We employed Arduino libraries to implement algorithms in the network controller, and the responders used to read from the analog IR sensors. Conversely, we used MicroPython modules to program the responders to read the 3-axis motion, vibration, and illuminance measurements. Using Arduino Software IDE version 2.1.0 (Arduino AG, Turin, Italy) and the QuickESPNow library for ESP-NOW network-level functions in version v0.8.1, we implemented an algorithm in the network controller to send broadcast calls (one-to-all) with its MAC address every 10 ms. The call consisted of a recurring incremental loop of 22 integer numbers. We then implemented a callback software function to read the responder’s data asynchronously, allocating the readings in a 22-element JSON object using the ArduinoJson library in version 6.21.2.

The library QuickESPNow was also applied to read from the analog IR sensors, utilizing a similar asynchronous callback function to read the controller’s call and MAC. Using an authentication check, we implemented a periodic loop to send unicast replies (one-to-one). Each reply consisted of a concatenated string composed of the responder ID and related readings. A similar approach was employed to read the 3-axis motion, vibration, and illuminance measurements using the Thonny 4.1.4 IDE (University of Tartu, Tartu, Estonia), Python 3.10.11 (Python Software Foundation, Wilmington, DE, USA) interpreter, and MicroPython modules Network, ESP-NOW, and Time. This implementation maintained the ESP-NOW protocol while leveraging Python’s capabilities for our custom Bluetooth-to-ESP-NOW interfaces.

**Figure 8:**
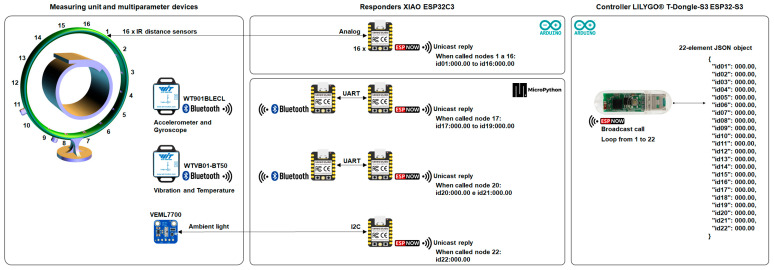
IIoT networking for monitoring belt rotation in pipe conveyors.

In the paired ESP32 interfaces, we utilized the MicroPython modules Time, Bluetooth, Struct, and Machine’s UART class. We used UART communication to transmit the readings from the Bluetooth-connected board (the sender) to the ESP-NOW networking board (the receiver). Using configuration data provided by the manufacturer, WitMotion Shenzhen, we implemented functions that handled events through callbacks, processed notifications to extract readings, and monitored the connection status. We also applied automatic reconnection mechanisms when communication timeouts were detected. Table 2 and Figure 8 illustrate the IIoT networking employed in this study for monitoring belt rotation in pipe conveyor applications.

#### 2.4.2. Enabling Technology 2: LLM-Assisted Computing

To provide a cost-effective and scalable alternative for supporting digitization in pipe conveyor applications, we employed LLM-assisted computing in this study. The reasons for using this approach include the flexibility of fine-tuning models as a service on remote servers, the continuous reduction in costs per token, and the capability of using a physical prototype, which provided a comprehensive training dataset, favoring model convergence during fine-tuning. To mitigate the known generalization issues in generating floating-point numbers, we selected GPT-4o-mini (OpenAI, San Francisco, CA, USA) as the base model for fine-tuning. We applied truncation to represent distance measures with five digits, consisting of three integer digits and two decimal places, using the findings from Yang et al. [50] as a reference for framing the experiment.

Following the implementation of the network layer, we programmed the ESP32 USB dongle to parse and print the 22-element JSON object through its serial port. We developed an algorithm in the application layer using Python IDLE 3.10.8 to historicize the measurement readings for LLM fine-tuning. We parsed the JSON-like string using the Orjson library (version 3.9.9) and historicized the data using the built-in CSV module. Additionally, we recorded the Cartesian coordinates from the function calculating the B/D ratio, which served as a calibration measure for the relative positioning between the physical prototype and the measurement apparatus.

To create the structured prompt for training and later inference purposes, we concatenated keywords with the 16 distance measurements (represented as 2-digit floating-point numbers), the overlap angle (also described as 2-digit floating-point numbers), and the belt’s outer edge position (represented as an integer). The prompt structure was designed to utilize the existing knowledge of the pretrained model. We incorporated application-specific keywords, such as “Measures,” “Angle,” and “Direction”, to imply a meaningful relationship between the input data and the desired output, thereby maximizing the use of the model’s attention mechanism. We also added special characters as suffixes and stop sequences (“#, @”) to format the LLM roles, as presented in Table 3. Finally, the complete string was formatted as a newline-delimited JSON (JSONL) for fine-tuning.

Next, we created a fine-tuning task on OpenAI servers. We used the base model GPT-4o mini, a cost-effective multimodal model released in July 2024, with a knowledge cutoff of October 2023, and intended for focused tasks with reduced latency. Aware of the potential generalization and loss function sensitivity issues caused by the adoption of continuous values in the prompt and completion strings, we validated our fine-tuned model using data collected from the measuring unit, rather than utilizing validation scores from the fine-tuning task. This was a preplanned decision-making process, given that the model was experimentally set to output floating-point numbers, for which differences were expected at the token level, which could lead to inaccurate validation calculations. After training, we implemented an algorithm in the application layer for assisted computing, utilizing API calls to our custom model hosted on OpenAI servers. During inference, the model was prompted with a temperature parameter of 0.2, a maximum response length of 200 tokens, and a request timeout of 200 ms.

#### 2.4.3. Enabling Technology 3: Human–Machine Interfacing

Finally, we implemented an HMI using Python IDLE 3.10.8 to deploy a fully functional sampling unit. The interface comprised command buttons to pause or resume sampling, enable or disable the LLM-assisted computing, historicize in a CSV file, toggle the belt’s edge position, and terminate the application. A status textbox periodically printed the timestamp along with the Cartesian coordinates from the function calculating the B/D ratio. Additionally, each toggled button triggered on-demand status messages in a textbox. We added numeric fields to be populated by the system user with the sample length (belt width) and thickness in mm. We then set the default values to 1700 and 20 mm, corresponding to the belt sample used in our experiment. A free-text field in the HMI allowed system users to annotate data with contextual information.

We plotted a polar chart and added a concentric point cloud to represent the measurement apparatus. This chart displays the 16 distance measures, a 1000-step spiral approximation, and two pointers representing the measured and predicted belt angles. The spiral was populated clockwise or anticlockwise based on the preset or predicted belt’s outer edge position. We also plotted a table with eleven readings, including the angle of the belt overlap (0–359.99°), rotation speed (in rpm), absolute belt turns, vibration velocity (in mm/s RMS), temperature (°C), illuminance (in lx), pipe belt diameter (in mm), overlap length (in mm), B/D ratio, the belt’s outer edge position (if undefined, right- or left-sided), and latency (in milliseconds).

**Figure 9:**
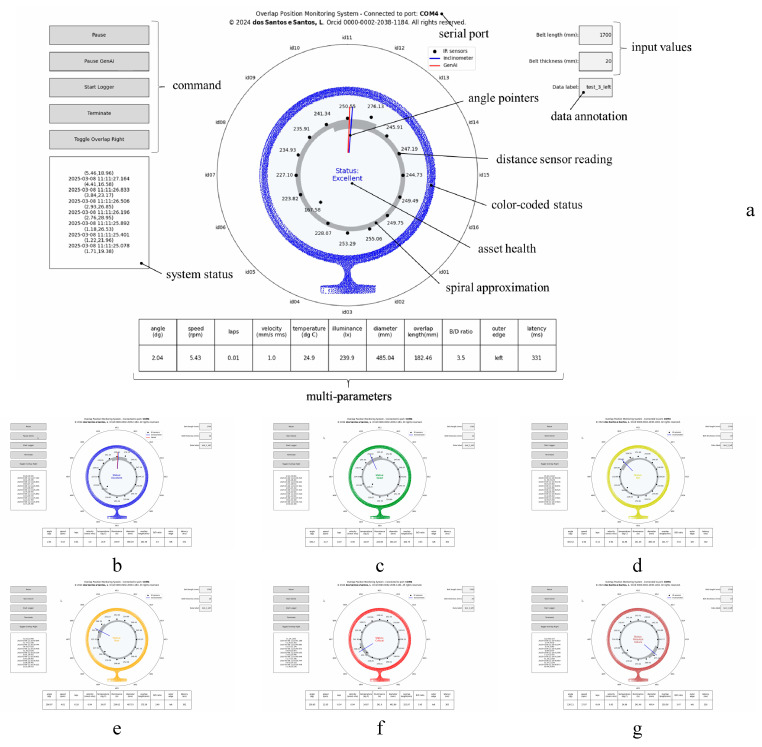
HMI key features (**a**) and color-coded statuses: excellent condition (**b**), good condition (**c**), fair condition (**d**), poor condition (**e**), critical condition (**f**), and above the adopted threshold for potential failure (**g**). Images (**b**–**g**) are displayed at a reduced size and resolution for demonstration purposes of the employed color code.

At startup, the program listed the available serial ports and automatically connected to the ESP32 USB dongle, populating its port number in the HMI. We added an asset health status label at the center of the point cloud. Both the image and status labels were color-coded as follows: excellent (blue), good (green), fair (yellow), poor (orange), critical (red), and potential failure (brown). Figure 9 illustrates the HMI used for information integration.

## 3. Results

**Figure 10:**
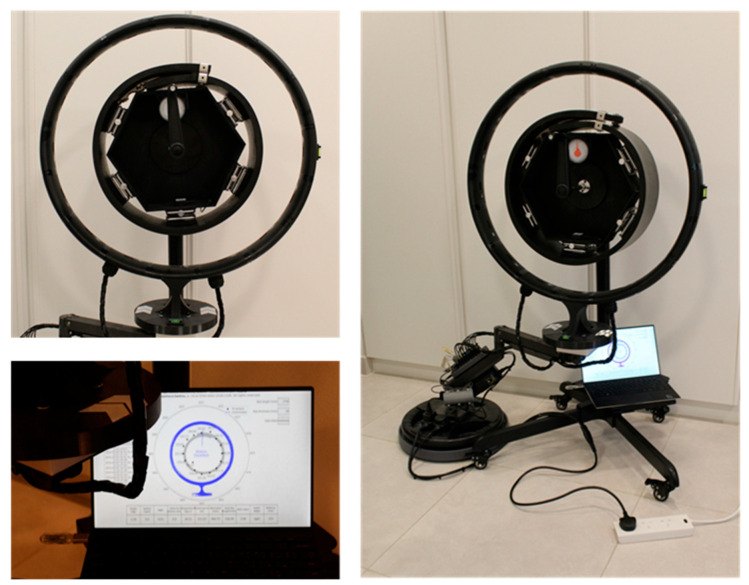
Advanced multiparameter system for monitoring belt rotation in pipe conveyor applications. HMI is displayed at a reduced size and resolution for general viewing purposes.

From our experimental setup, we collected 1217 samples, with 909 obtained in training mode and 308 processed in inference mode (after starting GenAI mode in the HMI). All samples were collected in a single trial, in which the outer edge of the belt was set to the left side. We applied multiple turns in the belt sample at different angular speeds to reproduce the belt rotation failure mode in various critical scenarios. The CSV file successfully historicized the program variables, labeled as follows: timestamp, distance measures (id01–id16), measured overlap angle (id17), angular speed (id18), number of turns (id19), vibration velocity (id20), room temperature (id21), illuminance (id22), belt width (id23), belt thickness (id24), belt outer edge (id25), pipe diameter (id26), overlap length (id27), B/D ratio (id28), predicted overlap angle (id29), calculated angular speed (id30), calculated number of turns (id31), predicted belt outer edge (id32), prompt, completion, labeling, tokens, and calibration. Figure 10 presents a commissioned and operational advanced multiparameter system for monitoring belt rotation in pipe conveyor applications.

**Figure 11:**
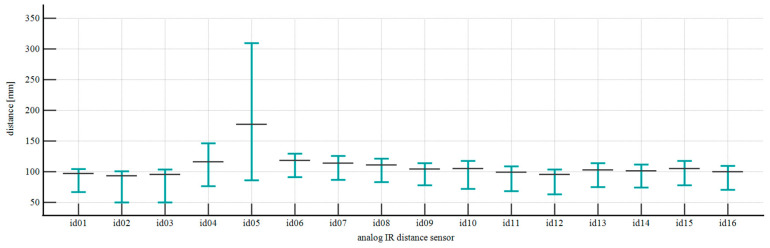
Measuring range for IR distance-measuring sensors.

During the development of the physical prototype, we employed the distance measurement characteristics provided in the manufacturer’s datasheet. Although the linearity region considered a range of 100 to 800 mm, the voltage readings enabled distance measurements from 50 mm using a polynomial transformation. To convert analog voltage readings (0–3.5 Vdc) to distance measurements (50–800 mm), we implemented a 10th-degree polynomial function. We then plotted the measuring range for the 16 IR sensors for graphical analysis, which revealed outlier behavior in sensor id05, as presented in Figure 11.

During inference, the mean vibration velocity was 1.0447 mm/s RMS with a 95% confidence interval of 1.0365–1.0529 mm/s RMS and a range of 0.8900–1.2400 mm/s RMS. The mean room temperature was 24.9444 °C, with a 95% confidence interval of 24.9418–24.9469 °C and a range of 24.8900–24.9900 °C. The mean illuminance was 246.4855 lx with a 95% confidence interval of 244.9672–248.0038 lx and a range of 231.6700–290.0700 lx.

The fine-tuning process utilized 400,869 trained tokens over three epochs with a batch size of 1, a learning rate multiplier of 1.8, and a seed value of 42. We used the base model GPT-4o mini with the self-supervised learning method. The training time was 46:02 min, comprising 2727 steps, and resulting in a final training loss of 0.7935. We then compared the predicted values obtained from the fine-tuned model with the measurements made during inference for validation. Additionally, we evaluated the latencies of the measurement apparatus in both training and inference modes. We also compared the calculated pipe diameter and overlap length values with the manual measurements from the physical prototype.

**Figure 12:**
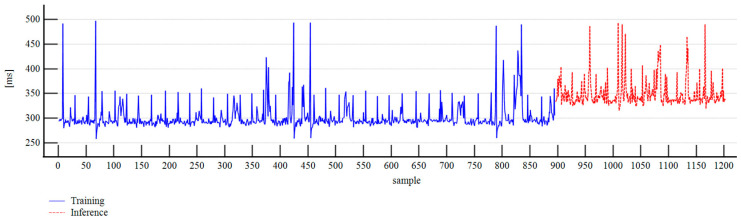
Latency of the measurement apparatus.

In the training mode, the mean latency was 301.6022 ms, with a 95% confidence interval of 299.9114 to 303.2931 ms, a standard deviation of 25.7734 ms, and a maximum value of 497 ms, observed in 895 samples. In inference mode, the mean latency increased to 350.1303 ms with a 95% confidence interval of 346.8893 to 353.3713 ms, a standard deviation of 28.8586 ms, and the highest value of 493 ms for 307 samples. Figure 12 presents the latency of the measurement apparatus in the training and inference modes.

Given the adoption of multithreaded execution and a weighted moving average to predict the angle of the belt overlap and position of the belt’s outer edge, we implemented a 10-cycle shift for data synchronization between the measured and predicted angles for method comparison purposes. For verification of the synchronization shift, we calculated the mean pairwise circular difference (absolute values) per cycle shift, obtaining a minimum of 10.5801° for 10 cycles. Furthermore, we employed the sine function to perform pairwise comparisons of angles and handle the transitions between 0° and 359.99°. Next, we used the Kolmogorov–Smirnov test to assess the normality of the data. The null hypothesis was rejected for the predicted and measured angles, with Kolmogorov–Smirnov statistics of 0.1041 and 0.1435, respectively. Next, we employed the nonparametric Wilcoxon signed-rank test for paired samples on the sine datasets. We found a statistically significant difference between the paired observations, with a test statistic of −7.087774 (*p* < 0.0001). Using the Hodges-Lehmann median difference, we obtained a bias of 0.0578, with a 95% confidence interval of 0.0417–0.0752. The standard deviation of the sine differences was 0.1471, with a precision of ±0.2883. The correlation coefficient was 0.9716, with a 95% confidence interval of 0.9645 to 0.9773. After applying the arcsine transformation, the bias corresponded to 3.3107° with a 95% confidence interval of 2.3899° to 4.3127°. The precision corresponded to ±16.7562°.

**Figure 13:**
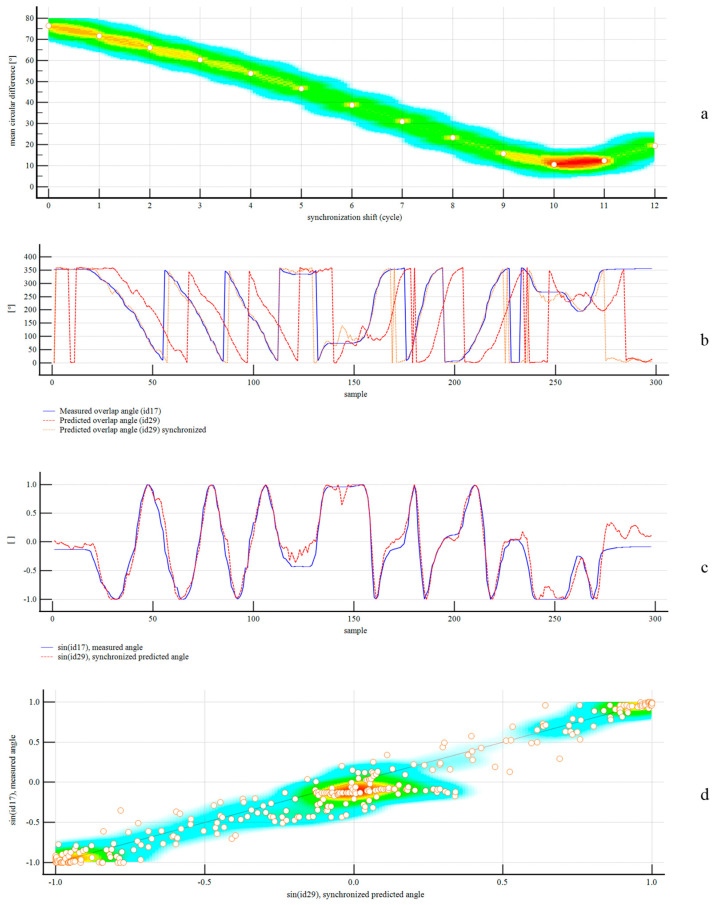
Results for angle measures: synchronization shift (**a**); predicted versus measured angle (**b**); sine function transformation for pairwise comparisons (**c**); scatter diagram of sine function transformation (**d**).

Figure 13 summarizes the results of the overlap angle prediction. In the trial, both training and predicted values for the belt outer-edge were left-sided and matched the actual values for all samples; however, the results were limited by the lack of right-sided sampling.

Next, we compared the calculated pipe diameter and overlap length with manual measurements from the physical prototype. We measured the diameter, considering the reference line of the hexagonal wooden frame in its three double apothems, and obtained 471, 479, and 472 mm. We then calculated the mean value of 474 mm, which was adopted as the test value for statistical analysis. We applied the Kolmogorov–Smirnov test to assess the normality of the data. The null hypothesis of normal distribution was rejected, with a test statistic of 0.0401. Consequently, we used the nonparametric one-sample Wilcoxon signed-rank test. The results showed a statistically significant difference between the sample median and the test value, with a test statistic of −30.217965 (*p* < 0.0001). Using the Hodges-Lehmann median difference, we obtained a bias of +13.3850 mm with a 95% confidence interval of 13.1800 to 13.5950 mm. The standard deviation was +3.6334 mm, and the measurement precision was ±7.2114 mm.

**Figure 14:**
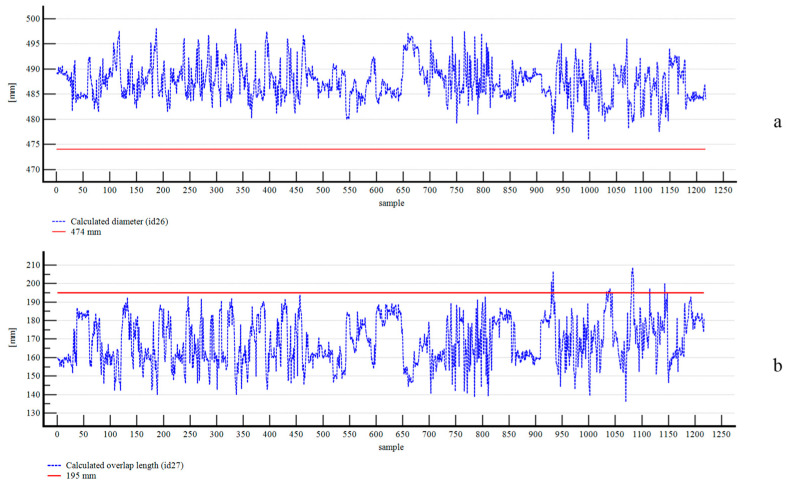
Results for the calculated pipe diameter (**a**) and overlap length (**b**).

Finally, we measured the overlap length in the physical prototype and obtained a value of 195 mm, which was adopted as the test value for statistical analysis. We applied the Kolmogorov–Smirnov test to assess the normality of the data. The null hypothesis of normal distribution was rejected, with a test statistic of 0.0920. Therefore, we used the nonparametric one-sample Wilcoxon signed-rank test. The results showed a statistically significant difference between the sample median and the test value, with a test statistic of −30.139555 (*p* < 0.0001). Using the Hodges-Lehmann median difference, we obtained a bias of −26.2750 mm with a 95% confidence interval of −27.0650 to −25.525 mm. The standard deviation was +12.8335 mm, and the measurement precision was ±25.1536 mm. Figure 14 summarizes the results for the calculated pipe diameter and overlap length.

**Figure 15:**
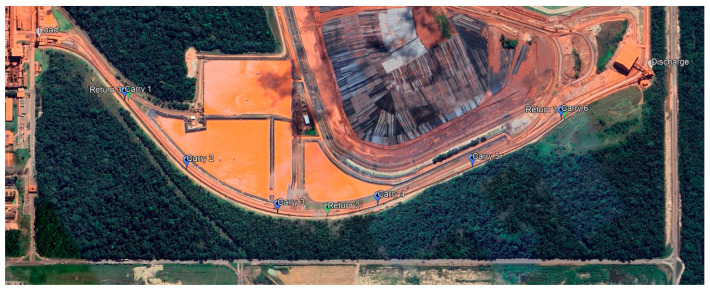
Recommended positioning of measuring units for digitization of belt rotation monitoring in a real-case pipe conveyor application.

Finally, we theorized the implementation of multiple sampling points. We applied the proposed model to a long-flight pipe conveyor that handles dried red mud. The conveyor has a developed length of 2213 m, with the most extended transition zone measuring 18 m. The nominal belt speed was 4.5 m/s, and the safe stop with a variable-frequency drive was set to 60 s. We assumed a critical belt rotation speed of 10 RPM, a half-turn as the admissible belt rotation ($n_{a}=0.5$), and a detection-response latency of 3.5 s to trigger a safe-state command. Adopting a criticality factor k of 1.5, our calculation suggested a unitary protection distance of 317.25 m and nine recommended measuring units (six on the carry flight and three on the return flight). Figure 15 illustrates the recommended positioning of the measuring units using our proposed methodology in a real-case scenario. Table 4 summarizes the calculations for the recommended number of measuring units.

## 4. Discussions

We designed and implemented a test rig in accordance with our proposed framework for digitalizing overlap monitoring in pipe conveyor belts. Our implementation expanded the findings of Jiang et al. [61], who investigated a data-model-service approach to integrate digital twin (DT) and IIoT, focusing on a device-DT-application. We added a physical prototype layer to support the alignment of the device-DT layer in a data-scarce application. The physical prototype functioned as intended, enabling the reproduction of multiple belt rotation scenarios that would be unachievable in a real-world application. The auto-buckle and safety c-clamps effectively held a fixed B/D ratio in the belt sample, and no abnormalities were observed during training or inference.

Belt-independent measurements were employed to map 16 points along the belt circumference. The predicted values using the fine-tuned LLM resulted in a measurement accuracy of 3.3107° ± 16.7562° for the overlap angle, influenced by an outlier in the analog IR measurement from network node number five. This also justified the variability at the precision level, as this fluctuation contributed to the model mapping patterns from the outlier node to the position of the belt’s inner edge. After assessing the overall performance and measuring range of similar sensors from the same batch, we concluded that this was a one-off case, for which on-demand replacement and retraining of the LLM are the recommended maintenance approaches for future industrial applications. When comparing the achieved precision level against a 180° belt rotation threshold at 10 RPM, representing a potential failure condition, the results remain promising. This is particularly significant given that existing solutions predominantly provide no traceability beyond the upper portion of the pipe belt shape, operating under the assumption of predominantly stable conditions that rarely reflect the operational stability of various pipe conveyor applications. This non-contact measurement approach supports the expanded interpretability of existing methods for measuring the forces of contact in pipe conveyors, as the experimental techniques developed at the Technical University of Kosice [52,62,63,64,65,66,67,68,69,70,71], once the belt rotation failure mode is a mechanical response to uneven contact forces.

Multiparameter processing, including vibration velocity, room temperature, and illuminance measurements, worked as intended for proof-of-concept. The variable-speed vibration motor simulated the excitation frequencies from the belt and idler rolls, as expected in real-world applications. In pipe conveyor applications, seasonal stops, prolonged downtime, and long-term storage of spare parts are common operational scenarios that can affect the condition of idler rolls [72]. Room temperature affects the viscoelastic behavior of the pipe belt, and IR sensors are sensitive to the direct spectrum of sunlight or halogen light.

The results of the pipe diameter calculations revealed a bias of +13.3850 mm with a precision of ±7.2114 mm. In turn, the findings for the overlap length calculations showed a bias of −26.2750 mm with a precision of ±25.1536 mm. These results are consistent with systematic differences that may require adjustment factors in future industrial implementations. The selected analog IR sensors utilize position-sensitive detectors based on the triangulation principle. Therefore, the radius of the measurement apparatus influences this calculation. A noise level was also expected, as the infrared-emitting diodes (IREDs) from neighboring devices remained powered while the apparatus was in operation. The magnitude of the difference in precision levels resulted from the employed calculations. The 1000-step spiral approximation for calculating the overlap length utilized predefined values for length (the belt width) and stroke (the belt thickness), as well as the calculated diameter. Therefore, the positive bias in the diameter measures resulted in an underestimation of the overlap length. When detecting unsafe states, this precision level is acceptable for industrial applications, considering the adopted threshold of a 15% reduction in belt diameter [33]. In this case study, this would represent a reduction of 71.1 mm, which would be approximately 10 times higher than the obtained precision level.

When monitoring absolute belt turns, we encountered latency and synchronization challenges, which have been highlighted in digitization studies by Frasheri et al. [34], and Liu et al. [35]. After switching from the training to inference mode, we observed that the mean latency increased from 301.6022 to 350.1303 ms. The implementation of multithreaded execution and a weighted moving average resulted in a 10-cycle delay. This approach was employed to enhance hallucination mitigation, compensate for the limitations of generating floating-point numbers using LLMs, and ensure inference stability. In practical terms, this resulted in approximately 3.5 s of processing time for detecting unsafe states related to belt turns. This is an admissible response time for long-flight pipe conveyors, which typically require several seconds to minutes to stop following a safe-stop request. These controlled stops prevent overfilling of the loading chutes, clogging of the unloading chutes, and a sudden surge in the forces of contact. Flywheels in drive pulleys and capstan brakes are commonly employed solutions for recovering the belt take-up [73,74,75].

Our controller-responder wireless network of ESP32 IIoT devices successfully enabled the acquisition of 22 system variables in a JSON-like string. The custom Bluetooth-to-ESP-NOW interfaces also facilitated information integration by enabling a direct connection with off-the-shelf Bluetooth measuring devices. We then explored the use of LLMs under controlled experimental conditions for measurement applications by directly predicting the overlap angle and position of the outer edge of the belt. We incorporated application-specific keywords to optimize the model’s attention mechanism. This approach is similar to that employed by Tao et al. [46] in their bearing fault diagnosis, where the attention mechanism was frozen during fine-tuning.

The fine-tuning process using the base model GPT-4o mini in the self-supervised learning method was aligned with the approach proposed by Richey Jr et al. [4] to address logistic applications with recurring human intervention and insufficient data. This also aligns with the AI-driven knowledge management framework presented by Massaro et al. [76], who proposed supervised learning to process sensor data allocated to individual machines. Sommer et al. [77] highlighted that many companies avoid or delay digitization in built environments due to cost, time, and resource constraints, including a lack of information technology expertise. In this sense, adopting proprietary models facilitates this integration, saving resources and expertise through intuitive and user-friendly interfaces. However, it is worth noting that several open-source models and fine-tuning approaches are now widely available, expanding the possibilities for local inference and custom solutions.

The adoption of low-cost and commoditized ESP32 IIoT devices also plays a relevant role in supporting the scalability of our proposed solution, and a similar economic approach has been successfully employed in the most diverse innovative applications, such as human movement detection in smart buildings [78] and the recording of geoscientific readings for geotechnical monitoring [79], among others. Further, the implemented HMI successfully provided multiparameter monitoring of the pipe belt while historicizing the training and inference data. The color-coded asset health status and 11 other system variables provided timely detection of unsafe states, demonstrating the feasibility of deploying a fully functional sampling unit.

Our measurement apparatus successfully achieved the target features outlined in our digitization framework, including measures of the relative and absolute positions of the belt overlap, the belt rotation speed, and the B/D ratio. The results demonstrated a promising advantage of the proposed method over existing publicly available, patented, and off-the-shelf solutions. To achieve large-scale industrial scalability, mesh coordination and consensus mechanisms are necessary between multiple IIoT units to ensure the traceability of unsafe states along the belt flight. Rožanec et al. [80] proposed a human-centered architecture for integrating artificial intelligence into industrial applications, including, among others, modules for forecasting and decision-making to be integrated with user interfaces, which aligns with our proposed human–machine interfacing. Further, integrating cloud-based LLM-assisted computing for processing field data requires a risk-based assessment to ensure data privacy and security. Alonso et al. [81] developed a six-step framework that encompasses aspects such as user authentication and authorization, protection of data during transfer and storage, mechanisms for traceability and system monitoring, and measures that ensure both security and privacy. In addition, adopting multiple sampling along the route will demand easy deployment during LLM retraining, ensuring measurement accuracy and maintainability.

## 5. Conclusions

We demonstrated the implementation of a digitization framework to enable large-scale industrial applications of overlap monitoring in pipe conveyor belts, comprising six technological principles. To apply these principles, we developed an intermediate physical twin that overcame the data and labeling scarcity associated with the belt rotation failure mode. Next, using a controller-responder wireless network of ESP32 IIoT devices, we implemented a belt-independent measurement apparatus with multiparameter capability and a controller-responder IIoT network. We also developed Bluetooth-to-ESP-NOW interfaces that enabled direct connection with off-the-shelf measuring devices. This was achieved by employing three enabling technologies. We then proposed key criteria for detecting unsafe states related to belt rotation in pipe conveyors and developed a mathematical model to calculate the recommended number of measuring devices. The results for multiparameter processing are summarized in Table 5.

Finally, we fine-tuned an autoregressive LLM using the base model GPT-4o mini with the self-supervised learning method to predict the angle of the belt overlap and belt outer-edge position. Application-specific keywords were incorporated to optimize the model’s attention mechanism and mitigate the limitations of LLMs with floating-point numbers. Table 6 outlines the results for latency during training and inference, and Table 7 summarizes the results for measurements of overlap angle, pipe diameter, and overlap length.

Using an edge layer in Python, which included an HMI, we monitored and historicized training and inference data, including data annotation capability. We successfully implemented a color-coded asset health status display and monitoring of eleven key readings, including the angle of the belt overlap (0–359.99°), rotation speed (in rpm), absolute belt turns, vibration velocity (in mm/s RMS), temperature (°C), illuminance (in lx), pipe belt diameter (in mm), overlap length (in mm), B/D ratio, the belt’s outer edge position (if undefined, right- or left-sided), and latency (in milliseconds). Finally, we achieved the research objective of developing a digitization framework for belt rotation monitoring in pipe conveyor applications.

## 6. Future Works

This study had certain limitations that should be addressed in future research. First, trials with the right-sided belt outer edge or different diameters may require increased LLM training or result in degraded precision levels. Second, the outlier sensor in the analog IR measurement potentially impacted the overall performance; therefore, on-demand replacement and retraining of the LLM are recommended maintenance approaches for future industrial applications.

For future studies, we recommend the following:

- Exploring open-source small language models to assess the cost–benefit of local inference for minimizing detection-response latency.
- Validating our theoretical model for multiple sampling points along pipe conveyor routes in real-world industrial scenarios.
- Expanding the training dataset to cover both right- and left-sided outer edges and different belt diameters.

In the future, we will focus on optimizing the balance between the detectability of unsafe states, implementation and operation costs, and latency to enhance the practical applicability of our digitization framework for monitoring belt rotation in pipe conveyors, further expanding industrial information integration.

## Figures and Tables

**Table 1:** Key criteria for detecting unsafe states related to belt rotation in pipe conveyors.

Criteria	Value	Diagnostic	Color Code
Belt rotation	Up to 15° (1/24 turn)	Excellent	⬤ Blue
	Between 15° and 30° (1/24 and 1/12 turns)	Good	⬤ Green
	Between 30° and 45° (1/12 and 1/8 turns)	Fair	⬤ Yellow
	Between 45° and 90° (1/8 and 1/4 turns)	Poor	⬤ Orange
	Between 90° and 180° (1/4 and 1/2 turns)	Critical	⬤ Red
	Greater than 180° (1/2 turn)	Potential Failure	⬤ Brown
Reduction in diameter	Greater than 15% of nominal		-
Overlap alternation	Greater than or equal to 1 event		-
Belt rotation speed	Greater than or equal to 10 RPM		-
Belt twisting	Greater than or equal to 360° (1 turn)	Functional Failure	-

**Table 2:** IIoT networking devices and variables.

Function		Physical Nodes	SoCs	Variables	Coding IDE	Measurement	Sensors	Accuracy	Response Time	IIoT Device	Communication
Controller		1	1	—	Arduino	—	—	—	—	LILYGO T-Dongle S3 ESP32-S3	ESP-NOW → USB
Responder	Analog IR sensors	16	16	16		Distance	GP2Y0A21YK0F	—	39 ms	XIAO ESP32C3	Analog → ESP-NOW
	3-axis motion meter	1	2	3	MicroPython	Angle	WT901BLECL	±0.05°	100 ms		Bluetooth → UART → ESP-NOW
						Angular speed					
						Turns					
	Vibration meter	1	2	2		Vibration velocity	WTVB01-BT50	±30 mm/s	100 ms		Bluetooth → UART → ESP-NOW
						Room Temperature					
	Light sensor	1	1	1		Illuminance	VEML 7700	±10%	600 ms		I^2^C → ESP-NOW
Total		19	22	22	—	—	—	—	—	—	—

**Table 3:** Prompt formatting for fine-tuning purposes.

Prompt Formatting	Sample from Measurement Apparatus
system	Your responses always will be in the format Angle:Float,Direction:Integer@.
user	Given the Measures [id01:97.32,id02:92.73,id03:77.91,id04:104.21,id05:130.30,id06:114.05,id07:110.97,id08:111.73,id09:105.32,id10:104.79,id11:100.39,id12:98.96,id13:105.97,id14:106.49,id15:107.35,id16:100.97], predict the Angle and Direction#
assistant	Angle:174.11,Direction:2@
JSONL	{“messages”: [{“role”: “system”, “content”: “Your responses always will be in the format Angle:Float,Direction:Integer@.”}, {“role”: “user”, “content”: “Given the Measures [id01:97.32,id02:92.73,id03:77.91,id04:104.21,id05:130.30,id06:114.05,id07:110.97,id08:111.73,id09:105.32,id10:104.79,id11:100.39,id12:98.96,id13:105.97,id14:106.49,id15:107.35,id16:100.97], predict the Angle and Direction#”}, {“role”: “assistant”, “content”: “Angle:174.11,Direction:2@”}]}

**Table 4:** Calculations for the recommended number of measuring units.

Variable	Value
Criticality factor k	1.5
Developed conveyor length L (m)	2213
Length of the most extended transition zone $T_{z}$ (m)	18
Nominal longitudinal speed of the belt $V_{b}$ (m/s)	4.5
Admissible number of turns $n_{a}$	0.5
Critical belt rotation speed $\omega_{r}$ (RPM)	10
Detection-response latency $\tau_{dr}$ (s)	3.5
Safe-stop time with controlled or inertial deceleration $\tau_{s}$ (s)	60
Unitary protection distance (m)	317.25
Recommended number of measuring units $n_{u}$	9
Devices on the carry flight (⅔)	6
Devices on the return flight (⅓)	3

**Table 5:** Summary of multiparameter processing.

Parameters	Mean	Range
Vibration velocity	1.0447 mm/s RMS	0.8900–1.2400 mm/s RMS
Room temperature	24.9444 °C	24.8900–24.9900 °C
Illuminance	246.4855 lx	231.6700–290.0700 lx

**Table 6:** Latency during training and inference.

Latency	Mean	Standard Deviation	Max	Samples
Training	301.6022 ms	25.7734 ms	497 ms	895
Inference	350.1303 ms	28.8586 ms	493 ms	307

**Table 7:** Results for belt rotation monitoring.

Measures	Bias	Precision
Overlap angle	3.3107°	±16.7562°
Pipe diameter	+13.3850 mm	±7.2114 mm
Overlap length	−26.2750 mm	±25.1536 mm

## Data Availability

The data supporting the findings of this study are not publicly available as they will be included in a forthcoming patent application.
